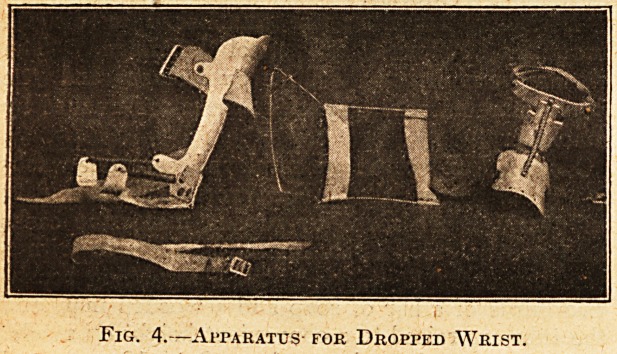# As Made at the Pavilion General Hospital, Brighton

**Published:** 1918-07-27

**Authors:** G. T. K. Maurice

**Affiliations:** Colonel A.M.S., O.C., Pavilion General Hospital


### TEMPORARY ARTIFICIAL LEGS

### As Made at the Pavilion General Hospital, Brighton.

### IV.—Details of Organisation.

I HOPE my description of the making of a leg will not make it seem a difficult and complicated operation to any who may be disposed to set themselves to give this boon to the maimed. It is not so in practice. I shall be glad to give anyone

FIG. 1.—DOUBLE AMPUTATION FIBRE RIGHT LEG, PLAISTER LEFT LEG. BELOW-KNEE CASE.

suited to the work who may desire to try it opportunity to see and practise the processes, and my skilful and painstaking head of the department will gladly explain every detail to those who earnestly desire the knowledge. A fortnight's steady work in the plaister-room would give more than the rudiments to one of a mechanical turn.

EACH DAY'S WORK.

In all work time is of importance. Each leg requires a maximum of four days. On the first day the mould is made, and by the evening it is dry enough to have the strengthening work done on it. The next day it will be dry enough to try on and trim and bind. The third day odds and ends of work will be done on it, and on the fourth day the man may wear it. The delay is due to the drying. This process varies with the plaister mixture, the thickness of the thickest parts of the bucket, the state of the weather, and the presence or absence of a fire in the plaister-room. If a good drying-room were provided, a leg could be made on one day and worn on the next. Working with one table and one operator, we can turn out eight or nine legs each day, but this is hard work. The operator is assisted by ladies who prepare bandages, etc., and a mechanic who does the carpentry and ironwork.

LEGS AT 2s. 6d. EACH.

I think I could organise a department with five tables, at each of which one good operator and two learner-assistants would work, and two mechanics to the whole department, not necessarily very skilled mechanics, which would turn out twenty-five to thirty legs a day—say seven thousand to eight thousand a year. But I have no idea of the cost of it. The legs we make here have cost about half-a-crown apiece, but all the workers are voluntary workers, ladies of Brighton and Hove and hospital patients, all the tools and machinery are lent, and a good deal of the material is scraps such as the bits of broken crutches. Other of the material—plaister, bandages, iron, and so forth,

FIG. 2.—MRS. PELLY'S PAPIER-MACHÉ SPLINT FOR SYME'S (OUTSIDE).

has been bought out of a sum of £150 the Red Cross gave us. Of that we have spent £32, and our June expenditure will be about £12, and we have made over 250 legs and a lot of "re-casts." That is to say, we have ripped off the old plaister when a stump has shrunk and made a new bucket on the old frame. The work began on January 29,

Previous articles appeared on July 6, 13 and 20, p. 343.

**Temporary Artificial Legs—(*continued*).**

1918, three weeks before I took command of the hospital, and a good deal had to be learnt, so that at first only two or three legs a week were made, but during the last two or three months skill has been improved, organisation has improved, and the

Fig. 2a.—Mrs. Pelly's Papier-mache Splint for Syme's (Inside).

output is getting larger. I think my estimate of seven thousand legs a year for a five-table department is not excessive. There is still only one table here, but I have now available three or four ladies who can do the work. Unfortunately, they cannot afford to give their time without other payment than my appreciation and the gratitude of the patients. If payment suitable for this skilled work were made to the workers, the cost of the legs would rise.

Standardised Peg Legs not Liked.

In the plaister-room recently we have been trying "the temporary leg—Erskine pattern"; this is a standardised peg leg. The men on whom we have tried it do not like it as well as the plaister leg. The great wooden ring is often not very comfortable. The idea of it is that by tightening a webbing strap the bucket can be made smaller as the stump shrinks. Unfortunately, stumps do not shrink equally all round, nor necessarily in a cone shape. It is easy to standardise peg legs, but you cannot standardise stumps.

The plaister leg is made to fit the man it is made for, as exactly as a first-class boot is made to fit by a first-class bootmaker who makes from measurement and hand-sews his goods. There is just the same sort of difference to the wearer between a plaister leg and a standard leg as there is between a boot made for a man and a ready-made pair. The Erskine leg has the advantage that it will outlast a plaister leg, and is there ready on the spot if it happens to fit the man trying it; but if it does not fit, it does not.

We have not yet found a man to fit the sizes above No. 6. Nos. 5, 4, and 3 seem the most useful. The sizes go from No. 1 to No. 8. No. 8 is the largest.

The plaister leg has the advantage that it considers individual anatomical differences and is adjusted on scientific knowledge and individual consideration of each case. The life of a plaister leg may be certainly as much as three months, but it is seldom they can be worn so long without re-making, as the alterations in the stumps that take place and continue when a man wears one are remarkably great. It is, however, very easy to re-make.

Fibre and Wood Legs.

At the Hove War Supply Depôt, Major Lightfoot makes a leg of fibre and wood which is very useful, and in one case has been preferred to the plaister-work leg by a man who has tried both. Usually, however, the plaister leg has been preferred, and as we specialise here on the plaister leg and are always busy at them, there is not much time for experiments. Though I do not think the fibre legs will ever give such good results as the plaister legs certainly do, I think they might be developed into a very useful article indeed (see Fig. 1).

Fig. 3.—Continuous Extension Apparatus for Flexed Stumps; very Effective. Made in the Plaister-Room.

Mrs. Pelly, at the same depôt, makes a papier-

(*Concluded on page 364.*)

### Temporary Artificial Legs (*Continued from page* 366).

mâché limb for Syme's amputations (see Figs. 2 and 2A). It is an excellent limb. The plaister-room has not yet evolved a plaister limb for these cases. Probably it could be done, but Mrs. Pelly's leg is so good, and the cases so comparatively rare, that they are always sent on to her. I had purposed to describe the making, but unfortunately, when writing, I found that a part of the process entails the use of a waterproofing solution which is a secret patent of the Willesden Canvas and Waterproofing Company, and is imparted to

FIG. 4.—APPARATUS FOR DROPPED WRIST.

the Hove Depôt under a pledge of secrecy as to its composition. While not approving the principle of keeping secret something which benefits the wounded for the profit or glorification of individuals or societies, we can but keep the condition. It is useless to publish an incomplete description.

At the Hove Depôt many clever bits of work are done, such as splints for wrist-drop and other special splints and odds and ends our surgeons require. They are beyond the scope of this paper, but I will slightly describe three instruments for mitigating the effects of wrist-drop (see Fig. 4). The left-hand side and right-hand side figures are photographs of instruments made by Private Maunder for his own hand. The left-hand apparatus consists of an arm-piece and a hand-piece hinged together and worn on the anterior aspect of the arm and hand. The steel springs hold the hand up and so enable use. The right-hand fixture is a similar instrument, but worn on the posterior aspect. The metal is pieces of aluminium alloyed and came from the tank of a wrecked airplane. The central instrument is made out of 28 inches of steel wire and two scraps of leather. The anterior leather goes at the back of the arm, the posterior over the front of the arm. The palm is supported on the longer portion of the cross wire, the projection on which goes between first finger and thumb. The spring is got by looping the wire in front of the anterior leather on each side. The instrument is light and efficient. It is an Italian idea. In the plaister-room a curious but effective plaister apparatus is made for the extension of flexed stumps (see Fig. 3). This also I shall not describe here, but I send photographs of some of the miscellaneous manufactures in hope that the Editor may approve them for publication.

In conclusion, I should like to thank all the workers who have assisted in the plaister-room, and especially the two patients, Privates Maunder and Edwards, for all the good work they have done. Maunder has worked indefatigably, despite the permanent handicap of his wounds, and Edwards has exercised his ingenuity so well that he has actually made himself a most excellent permanent leg out of odd scraps of material—fibre, leather, wood and so forth—such as we use in the plaister-room.

I also thank Mr. A. L. Stent for the excellent photographs he has taken to illustrate this paper.

It may interest readers to learn that the typescript of this article was done by Lance-Corporal F. E. Sharples, who has lost a leg, is wearing a plaister leg, and is a pupil in the Commercial Training Section of Queen Mary's Workshops.

G. T. K. MAURICE, Colonel A. M. S.,
O.C., Pavilion General Hospital.

## Figures and Tables

**Fig. 1. f1:**
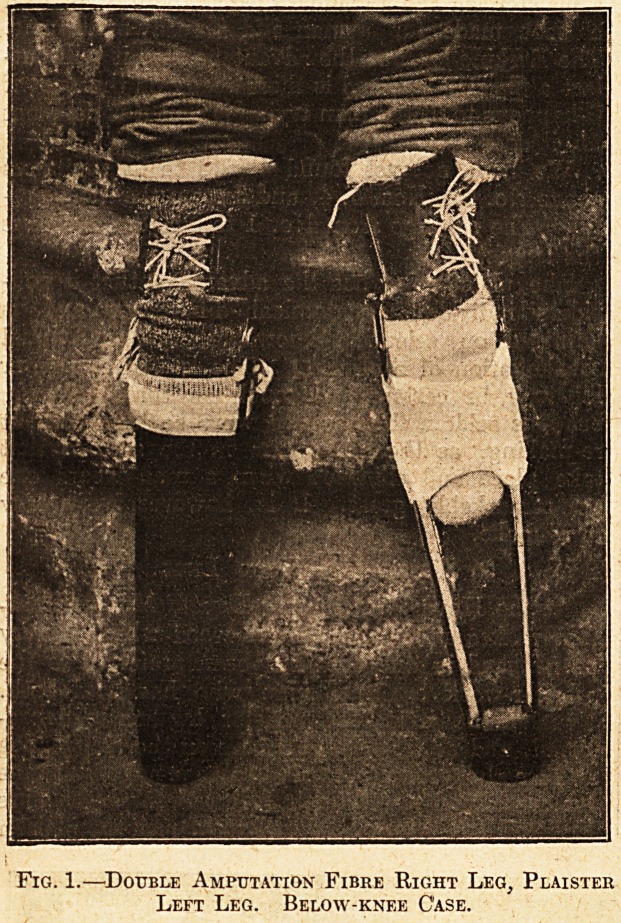


**Fig. 2. f2:**
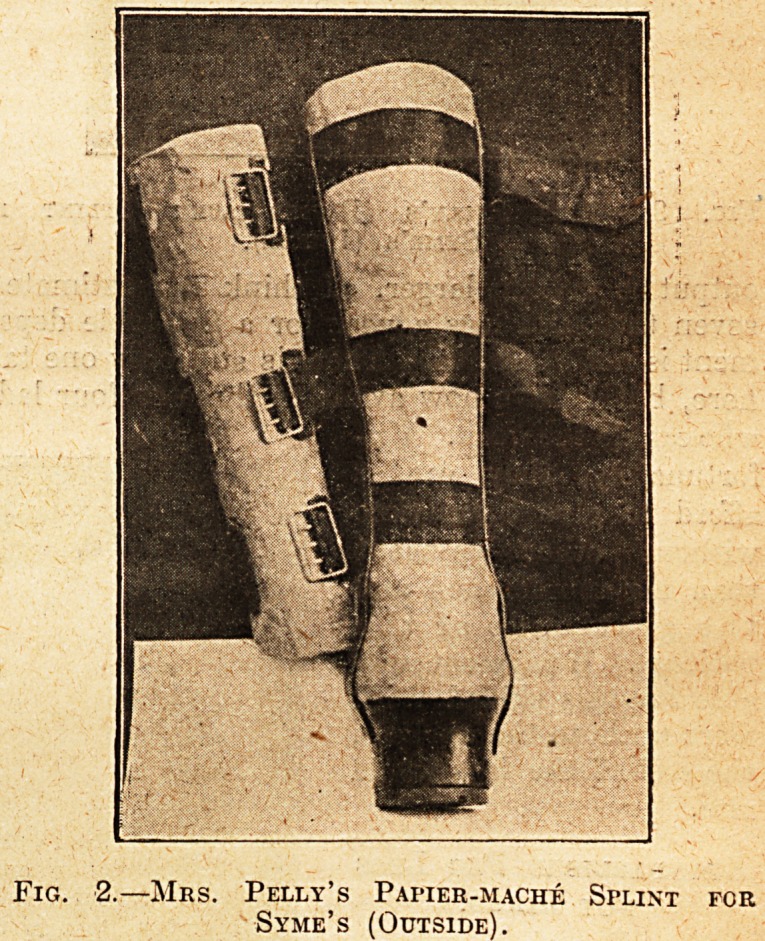


**Fig. 2A. f3:**
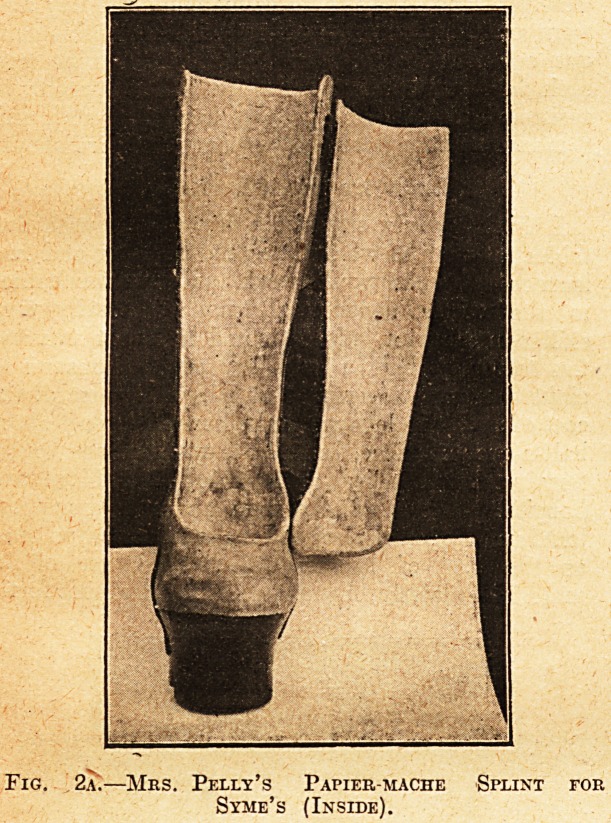


**Fig. 3. f4:**
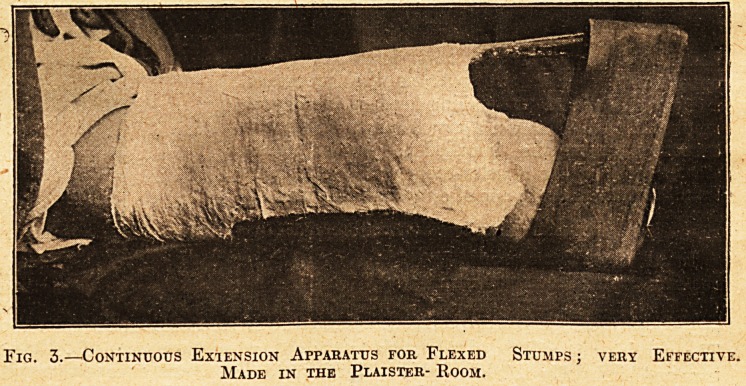


**Fig. 4. f5:**